# A fast and efficient Gibbs sampler for BayesB in whole-genome analyses

**DOI:** 10.1186/s12711-015-0157-x

**Published:** 2015-10-14

**Authors:** Hao Cheng, Long Qu, Dorian J. Garrick, Rohan L. Fernando

**Affiliations:** Department of Animal Science, Iowa State University, Ames, 50011 IA USA; Department of Statistics, Iowa State University, Ames, IA 50011 USA; Department of Mathematics and Statistics, Wright State University, Dayton, OH 45435 USA; Institute of Veterinary, Animal and Biomedical Science, Massey University, Palmerston North, New Zealand

## Abstract

**Background:**

In whole-genome analyses, the number *p* of marker covariates is often much larger than the number *n* of observations. Bayesian multiple regression models are widely used in genomic selection to address this problem of $p\gg n.$ The primary difference between these models is the prior assumed for the effects of the covariates. Usually in the BayesB method, a Metropolis–Hastings (MH) algorithm is used to jointly sample the marker effect and the locus-specific variance, which may make BayesB computationally intensive. In this paper, we show how the Gibbs sampler without the MH algorithm can be used for the BayesB method.

**Results:**

We consider three different versions of the Gibbs sampler to sample the marker effect and locus-specific variance for each locus. Among the Gibbs samplers that were considered, the most efficient sampler is about 2.1 times as efficient as the MH algorithm proposed by Meuwissen et al. and 1.7 times as efficient as that proposed by Habier et al.

**Conclusions:**

The three Gibbs samplers presented here were twice as efficient as Metropolis–Hastings samplers and gave virtually the same results.

## Background

In whole-genome analyses, the number *p* of marker covariates is often much larger than the number *n* of observations. Bayesian multiple regression models are widely used in genomic selection to address this problem of $p\gg n.$ The primary difference between these models is the prior assumed for the effects of the covariates. These priors and their effects on inference have been recently reviewed by Gianola [1]. In most Bayesian analyses of whole-genome data, inferences are based on Markov chains constructed to have a stationary distribution equal to the posterior distribution of the unknown parameters of interest [2]. This is often done by employing a Gibbs sampler where samples are drawn from the full-conditional distributions of the parameters [3].

It can be shown that in BayesA introduced by Meuwissen et al. [4], the prior for each marker effect follows a scaled *t* distribution [5]. However when the prior for the marker effect is specified as a *t* distribution, its full-conditional is not of a known form. Fortunately, this prior can also be specified as a normally distributed marker effect conditional on a locus-specific variance, which is given a scaled inverted chi-square distribution. When marginalized over the variance, this gives a *t* distribution for the marker effect [5]. Thus, the posterior for the marker effect would be identical under both these priors. The second form of the prior, however, is more convenient because it results in the full-conditional for the marker effect having a normal distribution.

BayesA is a special case of BayesB, also introduced by Meuwissen et al. [4], where the prior for each marker effect follows a mixture distribution with a point mass at zero with probability $\pi$ and a univariate-*t* distribution with probability $1-\pi$ [5]. When $\pi =0$, BayesB becomes BayesA. When the marker effect is non-null, as in BayesA, the second form of the prior leads to the full-conditional of the marker effect being normal. Nevertheless, Meuwissen et al. [4] used a Metropolis–Hastings (MH) algorithm to jointly sample the marker effect and the locus-specific variance because they argued that “the Gibbs sampler will not move through the entire sampling space” for BayesB. In their MH algorithm, they use the prior distribution of the locus-specific variance as the proposal distribution. When $\pi$ is high, the proposed values for the marker effect will be zero with high probability. Thus, for each locus, 100 cycles of MH algorithm were used in their paper, which makes BayesB computationally intensive. Habier et al. [6] used an alternative proposal, where the marker effect was zero with probability 0.5, that leads to a more efficient MH algorithm. For each locus, five cycles of MH were used to sample marker effects in this efficient MH method.

In this paper, we will show how Gibbs samplers without the MH algorithm can be used for the BayesB method. Recall that by introducing a locus-specific variance into BayesA, the full-conditional for the marker effects becomes normal. Similarly, in this paper we show that by introducing a variable $\delta _{j}$ in BayesB, indicating whether the marker effect for a locus is zero or non-zero, the marker effect and locus-specific variance can be sampled using Gibbs. We consider three different versions of the Gibbs sampler to sample each marker effect, locus-specific variance and its indicator variable $\delta _{j}$. The objectives of this paper are to introduce these samplers and study their performance.

## Methods

### Statistical methods

BayesB introduced by Meuwissen et al. [4] assumes that each locus-specific variance follows a mixture distribution. However, following Gianola [5], we prefer to specify the mixture at the level of the marker effect instead of the locus specific variance. In this formulation, the prior for the marker effect is a mixture with a point mass at zero and a univariate normal distribution conditional on $\sigma _{j}^{2}$:

$$\begin{aligned} \left( \alpha _{j}|\sigma _{j}^{2}\right)&{\left\{ \begin{array}{ll} =0 &{} \text {with probability}\,\pi \\ \sim N\left( 0,\sigma _{j}^{2}\right) &{} \text {with probability}\,\left( 1-\pi \right) , \end{array}\right. } \end{aligned}$$

where $\sigma _{j}^{2}$ follows a scaled inverted chi-square distribution and $\pi$ is treated as known. Employing the concept of data augmentation, it is convenient to write the marker effect $\alpha _{j}$ as $\alpha _{j}=\beta _{j}\delta _{j}$, where we introduce a Bernoulli variable $\delta _{j}$ with probability of success $1-\pi$ and normally distributed variable $\beta _{j}$ with mean zero and variance $\sigma _{j}^{2}$, which has a scaled inverted chi-square distribution. As shown below, Gibbs sampling can be used to draw samples for these unknowns.

#### Gibbs samplers for BayesB

Here, we present three Gibbs samplers for BayesB. The first is a single-site Gibbs sampler, where all parameters are sampled from their full conditional distributions. The second is a joint Gibbs sampler, where $\delta _{j},\beta _{j}$ are sampled from the joint full-conditional distribution $f\left( \delta _{j},\beta _{j}|\varvec{y},\mu ,\varvec{\beta }_{-j},\varvec{\delta }_{-j},\varvec{\xi },\sigma _{e}^{2}\right)$, where $\varvec{\xi }=\left[ \sigma _{1}^{2},\sigma _{2}^{2},\ldots ,\sigma _{k}^{2}\right]$, because $\delta _{j}$ and $\beta _{j}$ are highly dependent. Carlin and Chib [7] have shown that the prior used for parameters that are not in the model does not affect the Bayes factor. Thus, this prior, which they call a pseudo prior, can be chosen to improve mixing of the sampler. Following Carlin and Chib, the third sampler is a Gibbs sampler where a pseudo prior is used for $\beta _{j}$ when $\delta _{j}$ is zero. Godsill [8] has shown that the marginal posterior for a parameter in the model does not depend on the choice of pseudo priors. It has been suggested to choose the full conditional distribution for $\beta _{j}$ when it is in the model as the pseudo prior [7, 8]. This choice is justified by showing that using this pseudo prior is equivalent to sampling $\delta _{j}$ from $f\left( \delta _{j}|\varvec{y},\mu ,\varvec{\beta }_{-j},\varvec{\delta }_{-j},\varvec{\xi },\sigma _{e}^{2}\right)$ [8]. However, in BayesB, use of the exact full conditional distribution as the pseudo prior will require MH to sample $\sigma _{j}^{2}$ and $\sigma _{e}^{2}$. Thus in this paper, a distribution close to the full conditional is used.

#### BayesB model with data augmentation model

1 $$\begin{aligned} y_{i}&=\mu +\sum _{j=1}^{k}X_{ij}\beta _{j}\delta _{j}+e_{i}, \end{aligned}$$

where $y_{i}$ is the phenotype for individual i, $\mu$ is the overall mean, k is the number of SNPs, $X_{ij}$ is the genotype covariate at locus j for animal i (coded as 0, 1, 2), $\beta _{j}$ is the allele substitution effect for locus j, $\delta _{j}$ is an indicator variable and $e_{i}$ is the random residual effect for individual i.

#### Priors

The prior for $\mu$ is a constant. The prior for $e_{i}$ is $e_{i}|\sigma _{e}^{2}\overset{iid}{\sim }N\left( 0,\sigma _{e}^{2}\right)$ and $\left( \sigma _{e}^{2}|\nu _{e},S_{e}^{2}\right) \sim \nu _{e}S_{e}^{2}\chi _{\nu _{e}}^{-2}$. The prior for $\beta _{j}$ is $\beta _{j}|\sigma _{j}^{2}\sim N\left( 0,\sigma _{j}^{2}\right)$. The prior for $\left( \sigma _{j}^{2}|\nu _{\beta },S_{\beta }^{2}\right) \sim \nu _{\beta }S_{\beta }^{2}\chi _{\nu _{\beta }}^{-2}$. The prior for $\delta _{j}$ is

$$\begin{aligned} \left( \delta _{j}|\pi \right)&{\left\{ \begin{array}{ll} =1 &{} probability\,(1-\pi )\\ =0 &{} probability\,\pi . \end{array}\right. } \end{aligned}$$

#### Single-site Gibbs sampler

The full conditional distributions of $\mu ,\sigma _{j}^{2}$ and $\sigma _{e}^{2}$ are well-known [4, 9, 10]. Thus they are presented here without derivations. The full conditional of $\mu$ is a normal distribution with mean $\frac{1^{'}\mathbf {\mathbf {e_{\mu }}}}{n}$ and variance $\frac{\sigma _{e}^{2}}{n}$, where $\mathbf {e_{\mu }}=\mathbf {y}-\sum _{j=1}^{k}\mathbf {X}_{j}\beta _{j}\delta _{j}$ and *n* is the number of individuals. The full conditional distributions of $\sigma _{j}^{2}$ and $\sigma _{e}^{2}$ are both scaled inverted chi-square distributions; for $\sigma _{j}^{2}$, the scale parameter is $\frac{\left( \nu _{\beta }S_{\beta }^{2}+\beta _{j}^{2}\right) }{\nu _{\beta }+1}$ and the degrees of freedom parameter are $\nu _{\beta }+1$; for $\sigma _{e}^{2}$, the scale parameter is $\frac{\nu _{e}S_{e}^{2}+\varvec{e^{'}e}}{\nu _{e}+n}$and the degrees of freedom parameter are $\nu _{e}+n$, where $\mathbf {e}=\mathbf {y}-\mathbf {1}\mu -\sum _{j=1}^{k}\mathbf {X}_{j}\beta _{j}\delta _{j}$.

Next, we derive the full conditional distributions of $\beta _{j}$ and $\delta _{j}$. These full conditional distributions are proportional to the joint distribution of all parameters and $\mathbf {y}$, which can be written as

2 $$\begin{aligned}f\left( \varvec{y},\mu ,\varvec{\beta },\mathrm
{\mathbf {\varvec{\delta }}},\varvec{\xi },\sigma _{e}^{2}\right)
&\propto f\left( \varvec{y}\mid \mu ,\varvec{\beta },\delta
,\varvec{\xi }, \sigma _{e}^{2}\right)\,\,f\left( \varvec{\beta
}|\varvec{\xi }\right)\,\,f \left( \varvec{\delta
}\right)\,\,f\left( \varvec{\xi }\right)\,\,f\left( \sigma
_{e}^{2}\right) \nonumber \\ &\propto \left( \sigma
_{e}^{2}\right)^{-\frac{n}{2}}\exp \left( -\frac{\mathbf
{e}^{'}\mathbf {e}}{2\sigma _{e}^{2}}\right) {\displaystyle \prod
_{j=1}^{k}\frac{1}{\sqrt{2\pi }\left( \sigma _{j}^{2}\right)
^{\frac{1}{2}}}\exp \left(
-\frac{\beta _{j}^{2}}{2\sigma _{j}^{2}}\right) }\nonumber \\
&\quad\times\prod _{j=1}^{k}\pi ^{\left( 1-\delta _{j}\right)
}\left( 1-\pi \right) ^{\delta _{j}} \prod _{j=1}^{k}\frac{\left(
S_{\beta }^{2}\frac{\nu _{\beta }}{2}\right) ^{\frac{\nu _{\beta
}}{2}}}{\Gamma \left( \frac{\nu _{\beta }}{2}\right) }\left( \sigma
_{j}^{2}\right) ^{\left( -\frac{\nu _{\beta }+2}{2}\right) }\exp
\left( -\frac{\nu _{\beta }S_{\beta }^{2}}{2\sigma _{j}^{2}}\right)
\nonumber \\ & \quad\times\frac{\left( S_{e}^{2}\frac{\nu
_{e}}{2}\right) ^{\frac{\nu _{e}}{2}}}{\Gamma \left( \frac{\nu
_{e}}{2}\right) }\left( \sigma _{e}^{2}\right) ^{\left( -\frac{\nu
_{e}+2}{2}\right) } \exp \left( -\frac{\nu _{e}S_{e}^{2}}{2\sigma
_{e}^{2}}\right) .
\end{aligned}$$

The full conditional distribution of $\beta _{j}$ is now obtained by dropping factors that do not involve $\beta _{j}$, which gives

$$\begin{aligned}f\left( \beta _{j}|\varvec{y},\mu ,\beta _{-j},
\varvec{\delta },\varvec{\xi },\sigma _{e}^{2}\right) &\propto \,
f\left( \varvec{y}\mid \mu ,\varvec{\beta },\mathbf {\varvec{\delta
}},
\varvec{\xi },\sigma _{e}^{2}\right) \, f\left( \beta _{j}|\sigma _{j}^{2}\right) \\
&\propto \exp \left( -\frac{\mathbf {e}^{'}\mathbf {e}}{2\sigma
_{e}^{2}}\right) \exp \left( -\frac{\beta _{j}^{2}}{2\sigma
_{j}^{2}}\right) \\ &\propto\exp \left[ -\frac{\left(
\varvec{w}-\varvec{X}_{\mathbf {j}}\beta _{j}\delta
_{j}\right)^{'}\left( \varvec{w}-\mathbf {\varvec{X}}_{\mathbf
{\mathbf {j}}}\beta _{j}\delta _{j}\right) }{2\sigma
_{e}^{2}}\right] \exp \left( -\frac{\beta _{j}^{2}}{2\sigma
_{j}^{2}}\right) , \end{aligned}$$

where $$\varvec{w}=\varvec{y}-\varvec{1}\mu -{\displaystyle \sum _{j^{'}\ne j}}\varvec{X}_{j^{'}}\beta {}_{j^{'}}\delta _{j^{'}}$$. When $$\delta _{j}=1$$,

3 $$\begin{aligned}f\left( \beta _{j}|\varvec{y},\mu ,\varvec{\beta} _{-j},\varvec{\delta },\varvec{\xi },\sigma _{e}^{2}\right) & \propto \exp \left[ -\frac{\left( \varvec{w}-X_{j}\beta _{j}\right) ^{'}\left( \varvec{w}-X_{j}\beta _{j}\right) }{2\sigma _{e}^{2}}\right] \exp \left( -\frac{\beta _{j}^{2}}{2\sigma _{j}^{2}}\right) \nonumber \\ &\propto\exp \left[ -\frac{1}{2}\frac{\left( \beta _{j}-\frac{\mathbf{X}_{j}^{'}\varvec{w}}{c_{j}}\right) ^{2}}{\frac{\sigma _{e}^{2}}{c_{j}}}\right] \end{aligned}$$

where $c_{j}=\varvec{X_{j}^{'}X_{j}}+\frac{\sigma _{e}^{2}}{\sigma _{j}^{2}}$. Now, (3) can be recognized as the kernel of a normal distribution with mean $\frac{\mathbf{X}_{j}^{'}\varvec{w}}{c_{j}}$ and variance $\frac{\sigma _{e}^{2}}{c_{j}}$. When $\delta _{j}=0$,

$$\begin{aligned}f\left( \beta _{j}|\varvec{y},\mu ,\varvec{\beta} _{-j},\varvec{\delta },\varvec{\xi },\sigma _{e}^{2}\right) & \propto\exp \left( -\frac{\varvec{w}^{'}\varvec{w}}{2\sigma _{e}^{2}}\right) \exp \left( -\frac{\beta _{j}^{2}}{2\sigma _{j}^{2}}\right) , \end{aligned}$$

and dropping the factor $\left[ \exp \left( -\frac{\varvec{w}^{'}\varvec{w}}{2\sigma _{e}^{2}}\right) \right]$, which is free of $\beta _{j}$, gives

$$\begin{aligned} f\left( \beta _{j}|\varvec{y},\mu ,\beta _{-j},\mathrm {\mathbf {\delta }},\xi ,\sigma _{e}^{2}\right)&\propto \exp \left( -\frac{\beta _{j}^{2}}{2\sigma _{j}^{2}}\right) , \end{aligned}$$

which is the kernel of a normal distribution with null mean and variance $\sigma _{j}^{2}$. Thus,

$$\begin{aligned} p(\beta _{j}|ELSE)={\left\{ \begin{array}{ll} \sim N\left( \frac{\mathbf{X}_{j}^{'}\varvec{w}}{c_{j}},\frac{\sigma _{e}^{2}}{c_{j}}\right) &{} when\,\delta _{j}=1,\\ \sim N\left( 0,\sigma _{j}^{2}\right) &{} when\,\delta _{j}=0, \end{array}\right. } \end{aligned}$$

where ELSE stands for all the other parameters and y. This means when $\delta _{j}=1$, the sampling of $\beta _{j}$ is identical to that in BayesA; when $\delta _{j}=0$, $\beta _{j}$ is sampled from its prior. This is different from the original implementation of BayesB introduced by Meuwissen et al. [4].

Similarly, the full conditional distribution of $\delta _{j}$ can be obtained from (2) by dropping all factors free of $\delta _{j}$, which gives

$$\begin{aligned}\Pr \left( \delta _{j}=1\mid ELSE\right) &\propto f\left( \varvec{y}\mid \mu ,\varvec{\beta },\mathbf {\varvec{\delta }},\varvec{\xi },\sigma _{e}^{2}\right) \Pr \left( \delta _{j}=1\right) \\ & \propto\exp \left[ -\frac{\left( \varvec{w}-\varvec{X}_{\mathbf {j}}\beta _{j}\delta _{j}\right) ^{'}\left( \varvec{w}-\mathbf {\varvec{X}}_{\mathbf {\mathbf {j}}}\beta _{j}\delta _{j}\right) }{2\sigma _{e}^{2}}\right] \left( 1-\pi \right) \\ &\propto\exp \left[ -\frac{\left( \varvec{w}-X_{j}\beta _{j}\right) ^{'}\left( \varvec{w}-X_{j}\beta _{j}\right) }{2\sigma _{e}^{2}}\right] \left( 1-\pi \right) , \end{aligned}$$

$$\begin{aligned}\Pr \left( \delta _{j}=0\mid ELSE\right) & \propto f\left( \varvec{y}\mid \mu ,\varvec{\beta },\mathbf {\varvec{\delta }},\varvec{\xi },\sigma _{e}^{2}\right) \Pr \left( \delta _{j}=0\right) \\ &\propto\exp \left[ -\frac{\left( \varvec{w}-\varvec{X}_{\mathbf {j}}\beta _{j}\delta _{j}\right) ^{'}\left( \varvec{w}-\mathbf {\varvec{X}}_{\mathbf {\mathbf {j}}}\beta _{j}\delta _{j}\right) }{2\sigma _{e}^{2}}\right] \pi \\ &\propto\exp \left( -\frac{\varvec{w}^{'}\varvec{w}}{2\sigma _{e}^{2}}\right) \pi . \end{aligned}$$

Thus,

$$\begin{aligned}\Pr \left( \delta _{j}=1\mid ELSE\right) =\frac{(1-\pi )d_{j}}{(1-\pi )d_{j}+\pi \exp \left( -\frac{\varvec{w^{'}w}}{2\sigma _{e}^{2}}\right) }, \end{aligned}$$

where $$d_{j}=\exp \left[ -\frac{\left( \varvec{w}-\varvec{X_{j}}\beta _{j}\right) ^{'}\left( \varvec{w}-\varvec{X}_{j}\beta _{j}\right) }{2\sigma _{e}^{2}}\right]$$.

#### Joint Gibbs sampler

The same priors as in single-site Gibbs sampler are used here. The only difference is that $\delta _{j},\,\beta _{j}$ are sampled from their joint full conditional distribution, which can be written as the product of the full conditional distribution of $\beta _{j}$ given $\delta _{j}$ and marginal full conditional distribution of $\delta _{j}$:

$$\begin{aligned}f\left( \delta _{j},\beta _{j}|\varvec{y},\mu ,\varvec{\beta }_{-j},\varvec{\delta }_{-j},\varvec{\xi },\sigma _{e}^{2}\right) &= f\left( \beta _{j}|\delta _{j},\varvec{y},\mu ,\varvec{\beta }_{-j},\varvec{\delta }_{-j},\varvec{\xi },\sigma _{e}^{2}\right) \\ &\quad \times\Pr \left( \delta _{j}|\varvec{y},\mu ,\varvec{\beta }_{-j},\varvec{\delta }_{-j},\varvec{\xi },\sigma _{e}^{2}\right) . \end{aligned}$$

Thus, $\delta _{j}$ is first sampled from $f\left( \delta _{j}|\varvec{y},\mu ,\varvec{\beta }_{-j},\varvec{\delta }_{-j},\varvec{\xi },\sigma _{e}^{2}\right)$. Then $\beta _{j}$ is sampled from $f\left( \beta _{j}|\delta _{j},\varvec{y},\mu ,\varvec{\beta }_{-j},\varvec{\delta }_{-j},\varvec{\xi },\sigma _{e}^{2}\right)$, which is identical to the sampling of $\beta _{j}$ in BayesB with single-site Gibbs sampler. The marginal full conditional for $\delta _j$ can be written as:

4 $$\begin{aligned}f\left( \delta _{j}|\varvec{y},\mu ,\varvec{\beta }_{-j},\varvec{\delta }_{-j},\varvec{\xi },\sigma _{e}^{2}\right) &\propto f\left( \varvec{y}\mid \delta _{j},\mu ,\varvec{\beta }_{\varvec{-j}},\varvec{\delta }_{\varvec{-j}},\varvec{\xi },\sigma _{e}^{2}\right) \Pr \left( \delta _{j}\right) \nonumber \\ &\propto f\left( \varvec{w}\mid \delta _{j},\sigma _{j}^{2},\sigma _{e}^{2}\right) \Pr \left( \delta _{j}\right) , \end{aligned}$$

where $\varvec{w}=\varvec{y}-\varvec{1}\mu -{\displaystyle \sum _{j^{'}\ne j}}\varvec{X}_{j{}^{'}}\beta _{j^{'}}\delta _{j^{'}}=\varvec{e}+\mathbf {X}_{j}\beta _{j}\delta _{j}$. Now $f\left( \varvec{w}\mid \delta _{j},\sigma _{j}^{2},\sigma _{e}^{2}\right)$ is a multivariate normal distribution with mean $E\left( \varvec{e}+\varvec{X}_{j}\beta _{j}\delta _{j}\mid \delta _{j},\sigma _{j}^{2},\sigma _{e}^{2}\right)$ and variance $Var\left( \varvec{X}_{j}\beta _{j}\delta _{j}+\varvec{e}\mid \delta _{j},\sigma _{j}^{2},\sigma _{e}^{2}\right)$. When $\delta _{j}=1$, it becomes a multivariate normal with null mean and variance $\varvec{X}_{j}\varvec{X}_{j}^{'}\sigma _{j}^{2}+\varvec{I}\sigma _{e}^{2}$; when $\delta _{j}=0$, it becomes a multivariate normal with null mean and variance $\varvec{I}\sigma _{e}^{2}$. Thus, samples can be drawn using

5 $$\begin{aligned}&f\left( \delta _{j}=1|\varvec{y},\mu ,\varvec{\beta }_{-j},\varvec{\delta }_{-j},\varvec{\xi },\sigma _{e}^{2}\right) \nonumber \\ & \quad =\frac{h_{1}\Pr \left( \delta _{j}=1\right) }{h_{1}\Pr \left( \delta _{j}=1\right) +h_{0}\Pr \left( \delta _{j}=0\right) },\end{aligned}$$

6 $$\begin{aligned} = &\frac{1}{1+\frac{h_{0}Pr\left( \delta _{j}=0\right) }{h_{1}\Pr \left( \delta _{j}=1\right) }} \end{aligned}$$

where $h_{i}=f\left( \varvec{w}\mid \delta _{j}=i,\sigma _{j}^{2},\sigma _{e}^{2}\right)$. However, evaluating the multivariate normal distribution $f\left( \varvec{w}\mid \delta _{j},\sigma _{j}^{2},\sigma _{e}^{2}\right)$ is computationally intense. An efficient way is to use the univariate distribution of $\varvec{X}_{j}^{'}\varvec{w}$, which contains all the information from $\mathbf {\varvec{w}}$ about $\beta _{j}$, instead of the distribution of $\varvec{w}$, which is a multivariate. Thus, (5) can be written as

$$\begin{aligned}f\left( \delta _{j}=1|\varvec{y},\mu ,\varvec{\beta }_{-j},\varvec{\delta }_{-j},\varvec{\xi },\sigma _{e}^{2}\right) =  \frac{m_{1}\Pr \left( \delta _{j}=1\right) }{m_{1}\Pr \left( \delta _{j}=1\right) +m_{0}\Pr \left( \delta _{j}=0\right) }, \end{aligned}$$

where $m_{i}=f\left( \varvec{X_{j}^{'}w}\mid \delta _{j}=i,\mu ,\varvec{\beta }_{-j},\varvec{\delta }_{-j},\varvec{\xi },\sigma _{e}^{2}\right)$, $m_{1}$ is an univariate normal distribution with null mean and variance $\left( \varvec{X_{j}^{'}X_{j}}\right) ^{2}\sigma _{j}^{2}+\varvec{X_{j}^{'}X_{j}}\sigma _{e}^{2}$, and $m_{0}$ is an univariate normal distribution with null mean and variance $\varvec{X_{j}^{'}X_{j}}\sigma _{e}^{2}$.

#### Gibbs sampler with pseudo priors

Here, following Carlin and Chib [7], a pseudo prior is used for $\beta _{j}$ when $\delta _{j}$ is zero. They proposed to use the full conditional distribution of $\beta _{j}$ when $\delta _{j}=1$ as the pseudo prior for $\beta _{j}$ when $\delta _{j}=0$ [7, 8], which results in the prior for $\beta _{j}$ as

$$\begin{aligned}&\beta _{j}|\delta _{j} = \, {\left\{ \begin{array}{ll} \sim N\left( 0,\sigma _{j}^{2}\right) &{} \,\delta _{j}=1\\ \sim f\left( \beta _{j}|\delta _{j}=1,\varvec{y},\mu ,\varvec{\beta }_{-j},\varvec{\delta }_{-j},\varvec{\xi },\sigma _{e}^{2}\right) &{} \,\delta _{j}=0. \end{array}\right. } \end{aligned}$$

We show below that the posterior mean of the marker effect, $\alpha _{j}=\beta _{j}\delta _{j}$, does not depend on the pseudo prior. This posterior mean can be written as:

7 $$\begin{aligned}E\left( \beta _{j}\delta _{j}\mid \mathbf {y}\right) &= \, \sum _{\delta _{j}}\int \beta _{j}\delta _{j}f\left( \beta _{j},\delta _{j}\mid \varvec{y}\right) d\beta _{j}\nonumber \\ &= \frac{\sum _{\delta _{j}}\int \beta _{j}\delta _{j}f\left( \varvec{y}\mid \beta _{j},\delta _{j}\right) \, f\left( \beta _{j}\mid \delta _{j}\right) \Pr \left( \delta _{j}\right) d\beta _{j}}{f\left( \varvec{y}\right) }\nonumber \\ &= \frac{ \int \beta _{j}f\left( \varvec{y}\mid \beta _{j},\delta _{j}=1\right) \, f\left( \beta _{j}\mid \delta _{j}=1\right) \Pr \left( \delta _{j}=1\right) d\beta _{j}}{f\left( \varvec{y}\right) }. \end{aligned}$$

The numerator in (7) is free of the pseudo prior: $f\left( \beta _{j}\mid \delta _{j}=0\right)$. Furthermore, it can be seen from the model equation (1) that the value of $\mathbf {y}$ is free of $\beta _{j}$ when $\delta _{j}=0$. Thus, the marginal distribution of $\mathbf {y}$, the denominator of (7), does not depend on the pseudo prior, which is the distribution of $\beta _{j}$ when $\delta _{j}=0$. As both the numerator and denominator of (1) are free of the pseudo prior, it follows that the posterior mean of $\alpha _{j}$ does not depend on the pseudo prior for $\beta _{j}$.

We show here that, given this pseudo prior, the full conditional for $\delta _{j}$ is identical to the marginal full conditional distribution of $\delta _{j}$, $\Pr \left( \delta _{j}|\varvec{y},\mu ,\varvec{\beta }_{-j},\varvec{\delta }_{-j},\varvec{\xi },\sigma _{e}^{2}\right)$, which is used in the joint Gibbs sampler.

The full conditional probability of $\delta _{j}=1$ can be written as:

8 $$\begin{aligned} \Pr \left( \delta _{j}=1\mid ELSE\right)&=\frac{g_{1}}{g_{1}+g_{0}}\nonumber \\&=\frac{1}{1+\frac{g_{0}}{g_{1}}}, \end{aligned}$$

where

$$\begin{aligned} g_{i}&=Pr\left( \delta _{j}=i\mid ELSE\right) \\&\propto f\left( \varvec{y}\mid \delta _{j}=i,\beta _{j},\varvec{\delta}_{-j},\varvec{\beta}_{-j},\mu ,\varvec{\xi },\sigma _{e}^{2}\right) \\&\times \, f\left( \beta _{j}\mid \delta _{j}=i,\sigma _{j}^{2}\right) \times p\left( \delta _{j}=i\right) \\&\propto f\left( \varvec{w}\mid \delta _{j}=i,\beta _{j},\sigma _{e}^{2}\right) \\&\times \, f\left( \beta _{j}\mid \delta _{j}=i,\sigma _{j}^{2}\right) \times p\left( \delta _{j}=i\right) \end{aligned}$$

with $$i=0$$ or 1.

The ratio in the denominator of (8) is:

9 $$\begin{aligned} \frac{g_{0}}{g_{1}}&=f\left( \varvec{w}\mid \delta _{j}=0,\beta _{j},\sigma _{e}^{2}\right) \end{aligned}$$

10 $$\begin{aligned}&\times \frac{f\left( \beta _{j}\mid \delta _{j}=0\right) }{f\left( \varvec{w}\mid \delta _{j}=1,\beta _{j},\sigma _{e}^{2}\right) \times f\left( \beta _{j}\mid \delta _{j}=1,\sigma _{j}^{2}\right) }\nonumber \\&\times \frac{Pr\left( \delta _{j}=0\right) }{Pr\left( \delta _{j}=1\right) }. \end{aligned}$$

In the above equation, (9) is identical to $h_{0}$ in (5) used in the joint Gibbs sampler, because $f\left( \varvec{w}\mid \delta _{j}=0,\beta _{j},\sigma _{e}^{2}\right)$ is also a multivariate normal with null mean and variance $\varvec{I}\sigma _{e}^{2}$. We show below that (10) is identical to $h_{1}^{-1}$. Our proposed prior for $\beta _{j}$ when $\delta _{j}=0$ (the pseudo prior) can be written as:

11 $$\begin{aligned}p\left( \beta _{j}\mid \delta _{j}=0\right) &= f\left( \beta _{j}|\delta _{j}=1,\varvec{y},\mu ,\varvec{\beta }_{-j},\varvec{\delta }_{-j},\varvec{\xi },\sigma _{e}^{2}\right) \nonumber \\ &= f\left( \beta _{j}|\delta _{j}=1,\varvec{w},\sigma _{e}^{2},\sigma _{j}^{2}\right) \nonumber \\ &= \frac{f\left( \varvec{w}\mid \delta _{j}=1,\beta _{j},\sigma _{e}^{2}\right) f\left( \beta _{j}\mid \delta _{j}=1,\sigma _{j}^{2}\right) }{f\left( \varvec{w}\mid \delta _{j}=1,\sigma _{j}^{2},\sigma _{e}^{2}\right) }. \end{aligned}$$

After replacing the pseudo prior in (10) with (11), it becomes $$\begin{aligned} \frac{1}{f\left( \varvec{w}\mid \delta _{j}=1,\sigma _{j}^{2},\sigma _{e}^{2}\right) }, \end{aligned}$$ which is identical to $h_{1}^{-1}$. Thus, the ratio $\frac{g_{0}}{g_{1}}$ in (8) is identical to $\frac{h_{0}Pr\left( \delta _{j}=1\right) }{h_{1}Pr\left( \delta _{j}=0\right) }$ in (6), which proves the full conditional probability (8) of $\delta _{j}=1$, when the proposed prior is used, is identical to (6), the marginal full conditional probability of $\delta _{j}=1$, which is used in the joint Gibbs sampler.

Use of the exact full conditional distribution as the pseudo prior in BayesB, however, will require MH to sample $\sigma _{j}^{2}$ and $\sigma _{e}^{2}$. Thus, a distribution close to the full conditional is used here. Here, we use a normal distribution with mean $\frac{\mathbf{X}_{j}^{'}\varvec{w}}{\mathbf{X}_{j}^{'}\mathbf{X}_{j}+\widetilde{\lambda }}$and variance $\frac{\widetilde{\sigma _{e}^{2}}}{\mathbf{X}_{j}^{'}\mathbf{X}_{j}+\widetilde{\lambda }}$, where $\widetilde{\lambda }=\frac{\widetilde{\sigma _{e}^{2}}}{\widetilde{\sigma _{j}^{2}}}$, and $\widetilde{\sigma _{e}^{2}}$ and $\widetilde{\sigma _{j}^{2}}$ are means of the prior distributions for the residual and the marker effect variances, respectively.

Next, we will show the derivation of the full conditionals, which are proportional to the joint distribution of all parameters and $\mathbf {y}$. Here, the joint distribution of all parameters and $\varvec{y}$ can be written as:

12 $$\begin{aligned}f\left( \varvec{y},\mu ,\varvec{\beta },\mathrm {\mathbf {\varvec{\delta }}},\varvec{\xi },\sigma _{e}^{2}\right) &\propto f\left( \varvec{y}\mid \mu ,\varvec{\beta },\varvec{\delta },\varvec{\xi },\sigma _{e}^{2}\right) \, f\left( \varvec{\beta }\mid \varvec{\delta },\varvec{\xi }\right) \, f\left( \varvec{\delta }\right) \, f\left( \varvec{\xi }\right) \, f\left( \sigma _{e}^{2}\right) \end{aligned}$$

13 $$\begin{aligned} &\propto\left( \sigma _{e}^{2}\right) ^{-\frac{n}{2}}\exp \left( -\frac{\mathbf {e}^{'}\mathbf {e}}{2\sigma _{e}^{2}}\right) \end{aligned}$$

14 $$\begin{aligned} &\times{\displaystyle \prod _{j=1}^{k}}\left[ \frac{1}{\sqrt{2\pi }\left( \sigma _{j}^{2}\right) ^{\frac{1}{2}}}\exp \left( -\frac{\beta _{j}^{2}}{2\sigma _{j}^{2}}\right) \right] ^{\delta _{j}}\end{aligned}$$

15 $$\begin{aligned} &\times{\displaystyle \prod _{j=1}^{k} \left[ \frac{1}{\sqrt{2\pi }\left( \frac{\widetilde{\sigma _{e}^{2}}}{\varvec{X}_{j}^{'}\varvec{X}_{j}+\widetilde{\lambda }}\right) ^{\frac{1}{2}}} \right] }^{1-\delta _{j}}\end{aligned}$$

16 $$\begin{aligned} &\times{\displaystyle \prod _{j=1}^{k}\left\{ \exp \left[ -\frac{\left( \beta _{j}-\frac{\varvec{X}_{j}^{'}\varvec{w}}{\varvec{X}_{j}^{'}\varvec{X}_{j}+\widetilde{\lambda }}\right) ^{2}}{2\frac{\widetilde{\sigma _{e}^{2}}}{\varvec{X}_{j}^{'}\varvec{X}_{j}+\widetilde{\lambda }}}\right] \right\} }^{1-\delta _{j}}\end{aligned}$$

17 $$\begin{aligned} &\times\prod _{j=1}^{k}\pi ^{\left( 1-\delta _{j}\right) }\left( 1-\pi \right) ^{\delta _{j}}\end{aligned}$$

18 $$\begin{aligned}& \times\prod _{j=1}^{k}\frac{\left( S_{\beta }^{2}\frac{\nu _{\beta }}{2}\right) ^{\frac{\nu _{\beta }}{2}}}{\Gamma \left( \frac{\nu _{\beta }}{2}\right) }\left( \sigma _{j}^{2}\right) ^{\left( -\frac{\nu _{\beta }+2}{2}\right) }\exp \left( -\frac{\nu _{\beta }S_{\beta }^{2}}{2\sigma _{j}^{2}}\right) \end{aligned}$$

19 $$\begin{aligned} &\times\frac{\left( S_{e}^{2}\frac{\nu _{e}}{2}\right) ^{\frac{\nu _{e}}{2}}}{\Gamma \left( \frac{\nu _{e}}{2}\right) }\left( \sigma _{e}^{2}\right) ^{\left( -\frac{\nu _{e}+2}{2}\right) }\exp \left( -\frac{\nu _{e}S_{e}^{2}}{2\sigma _{e}^{2}}\right) . \end{aligned}$$

It is easy to see that the full conditional distribution of $\sigma _{e}^{2}$, which does not involve $\delta _{j}$, is the same as that in the single-site Gibbs sampler. Although $\mu$ also appears in $\varvec{w}$ in (16), (16) has no effect on the full conditional of $\mu$ because the columns of *X*, which are always centered, are orthogonal to the column vectors of ones so that $\varvec{X_{j}^{'}}\mathbf {1}\mu =\mathbf {0}$. Thus, the full conditional of $\mu$ is the same as that in the single-site Gibbs sampler. When $\delta _{j}=1$, the full conditional distribution of $\beta _{j}$ is identical to that in the single-site Gibbs sampler. When $\delta _{j}=0$, (16) is the only part that includes $\beta _{j}$. Thus the full conditional distribution of $\beta _{j}$ is:

$$\begin{aligned} p(\beta _{j}|ELSE)={\left\{ \begin{array}{ll} \sim N\left( \frac{\mathbf{X}_{j}^{'}\varvec{w}}{\varvec{X_{j}^{'}X_{j}}+\frac{\sigma _{e}^{2}}{\sigma _{j}^{2}}},\frac{\sigma _{e}^{2}}{\varvec{X_{j}^{'}X_{j}}+\frac{\sigma _{e}^{2}}{\sigma _{j}^{2}}}\right) &{}\delta _{j}=1,\\ \sim N\left( \frac{\mathbf{X}_{j}^{'}\varvec{w}}{\varvec{X_{j}^{'}X_{j}}+\widetilde{\lambda }},\frac{\widetilde{\sigma _{e}^{2}}}{\varvec{X_{j}^{'}X_{j}}+\widetilde{\lambda }}\right) &{}\delta _{j}=0. \end{array}\right. } \end{aligned}$$

When $\delta _{j}=1$, the full conditional distribution of $\sigma _{j}^{2}$ is the same as that in the single-site Gibbs sampler. When $\delta _{j}=0$, (18) is the only part that contains $\sigma _{j}^{2}$, which means it should be sampled from its prior. Thus when $\delta _{j}=1$, the full conditional distribution of $\sigma _{j}^{2}$ is a scaled inverted chi-square distribution with scale parameter $\frac{\nu _{\beta }S_{\beta }^{2}+\beta _{j}^{2}}{\nu _{\beta }+1}$ and degrees of freedom parameter $\nu _{\beta }+1$; when $\delta _{j}=0$, it is a scaled inverted chi-square distribution with scale parameter $S_{\beta }^{2}$ and degrees of freedom parameter $\nu _{\beta }$. The full conditional distribution of $\delta _{j}$ can be obtained from the joint distribution of all parameters and $\mathbf {y}$ by dropping all factors free of $\delta _{j}$, which gives

$$\begin{aligned}&\Pr \left( \delta _{j}=1\mid ELSE\right) \\ &\quad\propto f\left( \varvec{y}\mid \mu ,\varvec{\beta },\mathbf {\varvec{\delta }},\varvec{\xi },\sigma _{e}^{2}\right) \Pr \left( \delta _{j}=1\right) \, f\left( \beta _{j}\mid \delta _{j}=1\right) , \end{aligned}$$

$$\begin{aligned}&\Pr \left( \delta _{j}=0\mid ELSE\right) \\ &\quad\propto f\left( \varvec{y}\mid \mu ,\varvec{\beta },\mathbf {\varvec{\delta }},\varvec{\xi },\sigma _{e}^{2}\right) \Pr \left( \delta _{j}=0\right) \, f\left( \beta _{j}\mid \delta _{j}=0\right) . \end{aligned}$$

Compared to the full conditional distributions for $\delta _{j}$ in the single-site Gibbs sampler, the difference is the extra factor $f\left( \beta _{j}\mid \delta _{j}=1\right)$ and $f\left( \beta _{j}\mid \delta _{j}=0\right)$, because they cannot be canceled out as in the single-site Gibbs sampler. Thus,

$$\begin{aligned}&\Pr \left( \delta _{j}=1\mid ELSE\right) \\ =&\frac{(1-\pi )d_{j}f\left( \beta _{j}\mid \delta _{j}=1\right) }{(1-\pi )d_{j}f\left( \beta _{j}\mid \delta _{j}=1\right) +\pi \exp \left( -\frac{\varvec{w^{'}w}}{2\sigma _{e}^{2}}\right) f\left( \beta _{j}\mid \delta _{j}=0\right) }, \end{aligned}$$

where $$f\left( \beta _{j}\mid \delta _{j}=1\right) =\frac{1}{\sqrt{2\pi }\left( \sigma _{j}^{2}\right) ^{\frac{1}{2}}}\exp \left( -\frac{\beta _{j}^{2}}{2\sigma _{j}^{2}}\right)$$ and $$f\left( \beta _{j}\mid \delta _{j}=0\right) =\frac{1}{\sqrt{2\pi }\left( \frac{\widetilde{\sigma _{e}^{2}}}{X_{j}^{'}X_{j}+\widetilde{\lambda }}\right) ^{\frac{1}{2}}}\exp \left[ -\frac{\left( \beta _{j}-\frac{X_{j}^{'}\varvec{w}}{X_{j}^{'}X_{j}+\widetilde{\lambda }}\right) ^{2}}{2\frac{\widetilde{\sigma _{e}^{2}}}{X_{j}^{'}X_{j}+\widetilde{\lambda }}}\right]$$.

### Data

Real genotypic data and simulated phenotypic data were used here to compare BayesB using MH, efficient MH or the three different Gibbs samplers as described above. The genotypic data included 3961 individuals with 55,734 SNPs. The heritability of the simulated trait was 0.25. The training data contained 3206 individuals and the remaining individuals were used for testing. A chain of length of 50,000 was used to estimate parameters of interest. Prediction accuracies were calculated using different samplers. The effective sample sizes [11], which estimate the number of independent samples from a chain, were calculated for $\sigma _{e}^{2}$ to compare convergence rates for different methods. Computing time for different methods with the same number of iterations were also compared.

## Results

The number of effective samples per second of computing time was obtained for BayesB using MH, efficient MH or the three different Gibbs samplers. These three Gibbs samplers were almost twice as efficient as Metropolis–Hastings (Table 1). The prediction accuracies for different samplers, which are calculated as the correlation between estimated breeding values and simulated phenotypes, are all equal to 0.296. Posterior means of $\mu$ for these four samplers are all equal to 2.508. Posterior means of $\sigma _{e}^{2}$ for different samplers are almost equal, ranging from 0.955 to 0.957.

**Table 1:** Efficiency of alternative MCMC samplers for BayesB

	Alternative MCMC samplers				
	MH	Efficient MH	Single-site Gibbs	Joint Gibbs	Gibbs with pseudo prior
Computing time	90,009	70,714	52,452	44,726	47,043
Effective sample size	25,262	24,588	24,684	26,757	25,036
Effective samples/s	0.280	0.347	0.471	0.598	0.532

Efficiency of alternative MCMC samplers for BayesB. Results are given for the computing time in seconds to obtain 50,000 samples, effective sample size and effective samples/s for BayesB using Metropolis–Hastings (MH), single-site Gibbs sampler, joint Gibbs sampler and Gibbs sampler with pseudo priors

## Discussion

In the joint Gibbs sampler, $\delta _{j}$ and $\beta _{j}$ are sampled jointly, which addresses the problem of dependence between $\delta _{j}$ and $\beta _{j}$. Thus, the joint sampler had the largest effective sample size. However, in the single-site Gibbs sampler, $\delta _{j}$ and $\beta _{j}$ are sampled from their full conditionals, and thus due to the dependence between $\beta _{j}$ and $\delta _{j}$, the single-site Gibbs sampler had the smallest effective sample size. These differences in effective sample size, however, were small.

In the Gibbs sampler with pseudo priors, $\beta _{j}$ and $\delta _{j}$ are also sampled from their full conditionals. Recall that we have shown that the posterior mean of the marker effects does not depend on the pseudo prior. Furthermore, Godsill [8] has shown that the marginal posterior for parameters in the model do not depend on the pseudo prior, which is the prior for $\beta _{j}$ when $\delta _{j}=0$. As suggested by Carlin and Chib [7], when the full conditional distribution of $\beta _{j}$ when $\delta _{j}=1$ is chosen to be the pseudo prior, we have shown that the samples of $\beta _{j}$ and $\delta _{j}$ are identically distributed to those from the joint Gibbs sampler. Thus, the Gibbs sampler with pseudo priors will have a similar effective sample size as the joint Gibbs sampler.

However, when the full conditional distribution for $\beta _{j}$ when $\delta _{j}=1$ is used as the pseudo prior in BayesB, the full conditional distributions of $\sigma _{e}^{2}$ and $\sigma _{j}^{2}$ are not of known forms because $\sigma _{e}^{2}$ and $\sigma _{j}^{2}$ are in the pseudo prior for the marker effect. In contrast to BayesB, in the model used by Godsill [8] to justify the use of full conditional distributions as the pseudo priors, for simplicity, hyper-parameters such as $\sigma _{e}^{2}$ were omitted [8]. Here, we have replaced $\sigma _{e}^{2}$ and $\sigma _{j}^{2}$ in the pseudo prior with constants such that the full conditionals for $\sigma _{e}^{2}$ and $\sigma _{j}^{2}$ have scaled inverted chi-square distributions. This modification will give a pseudo prior whose distribution is close to that of the full conditional. In the Gibbs sampler with this pseudo prior, the effective sample size was smaller than in the joint Gibbs sampler but still larger than in the single-site Gibbs sampler.

## Conclusions

When a MH algorithm is used to jointly sample the marker effect and the locus-specific variance, the BayesB method is computationally intensive. After introducing a variable $\delta _{j}$, indicating whether the marker effect for a locus is zero or non-zero, the marker effect and locus-specific variance can be sampled using Gibbs sampler without MH. Among the Gibbs samplers that were considered here, the joint Gibbs sampler is the most efficient. This sampler is about 2.1 times as efficient as the MH algorithm proposed by Meuwissen et al. [4] and 1.7 times as efficient as that proposed by Habier et al. [6].
